# The Image as Language: The Creation and the Use of the Visual Message by Young University Students in Their Communicative Social Activity

**DOI:** 10.3389/fpsyg.2022.944187

**Published:** 2022-07-07

**Authors:** Alfredo Ramón-Verdú, José Víctor Villalba-Gómez

**Affiliations:** Department of Plastic, Musical, and Dynamic Expression, University of Murcia, Murcia, Spain

**Keywords:** visual language, visual communication, image, interpretation of images, photography, visual narrative

## Abstract

Visual language, taken from the creation and reception of image perspectives, is ever-present in mediatized societies. With an interest in knowing what the experience of this is, a study is carried out in a university context, in the Faculty of Education at the University of Murcia, with the participation of 321 young students. The main objective of this study is to delve into the visual message, as a universal language in its productive and interpretative aspects, within a context marked by technology and the large-scale creation and use of images. The investigation is carried out by starting with a typical target performance, an individual, objective questionnaire which included three dimensions: the use of photographs, the use of graphic images, and the level of reflection and veracity of the image. A Principal Components Analysis (PCA) is carried out, which gives rise to a new conceptual organization after the Oblimin rotation based on the underlying variables. The data shows significant differences depending on the educational interests, a steady decrease in the use of images as ages increase, as well as less use of the image as a language among participants who are men. Men also make fewer requests for permission and transfer of permission, which are for the use of their own image by third parties. There is also evidence of a greater social value given to the image by women, as well as greater reflection and questioning of the message over the thirties, also by women.

## Introduction

Photographic, analogical, and digital techniques are the result of obtaining lasting images and capturing the action of light on sensitive mediums. The very approximate representation of the reasons for, or the types of scenarios or contexts photographed, ultimately depends on the interpretation of the observer, conditioned by their own experience and culture. The images captured represent visual ideas that make up a language, a way of communicating, through creations with dialogic intentionality, on the basis of plastic resources similar to those of graphic design, as regards the compositive elements and the message, making a particular visual message act as a visual text, a transmitter of concepts and ideas.

The image, the visual culture, and the study of this in education is something which has been delved deeper into, on a large scale since the end of the XX and beginning of the XXI centuries, with great emphasis on the artistic educational aspect for the comprehension of visual culture (Hernández, 1996). In this sense, the possibilities images and their communicative aims provide, depend on whether they are from a creative perspective or if they take on a receptive role in which the message is interpreted. In both cases, an idea is constructed from the particularities of visual language (Berger, 2005, p. 41). There has always been much interest in studying visual semantics and the mechanisms which encourage the use of images, which can be seen in studies such as those of Domínguez-Toscano (1996) and Ferradini and Tedesco-Prieto (1997), on the use of advertising images for teaching and didactic purposes. One of the areas of interest is based on the education of what is seen (Bustamante-Bohórquez et al., 2008), the critical reading of the image (Revuelta-Blanco, 2008; Ubreva-Amor, 2008), and studies of the analysis of the representations of reality (Aparici et al., 2009). With respect to this, we have seen that the image has become a tool in a pedagogy that transforms (Subirana, 2015; De-Andrés-del-Campo et al., 2016; Huerta, 2021a) when approached from a consumerist and creative point of view. From a didactic point of view, during the process of creating an image, narratives are created in cognitive development in processes such as those of language, creativity, reasoning, or organization, in different areas such as the denoted and the connoted, which have learning effects on the creating person and on the context in which it takes place. In this way, visual language takes up its position at a level of transporter of ideas and concepts which need a dialogue, reflection, and interpretation. This tendency has been completely renewed nowadays with the staggering advance in technology.

When looking at the creation of images as a language and as a detonating action in the communicative process, there are investigations in which the field of arts education has been studied more in-depth and in human capabilities (Dondis, 2002; Manifold, 2009; Marcellán-Baraze et al., 2013; González et al., 2015), when investigating visual literacy in order to improve students' competences (Mesquita-Romero et al., 2022). Using images is very much related to the expectations and interests of the individual, which limits their reading and interpretation, as these would have been previously conditioned, having a certain influence on the daily and professional tasks of those who use the images (Marcellán-Baraze and Agirre-Arriaga, 2008; Marzal-Felici and Soler-Campillo, 2011). Besides, there is another set of limitations that affects the interpretation and visual reading, as demonstrated in studies by Casper et al. (2018) and Tallon-Miles et al. (2021).

In the context in which these communicative competencies develop, both formal and non-formal spaces come together in the daily space (Marcellán et al., 2014). This means that part of the creative activity is going to take place outside the educational scope, being heavily influenced by the social context of development, where varied languages of expression are interrelated, which are assiduously reflected in the different artistic manifestations. The creation of visual experiences predominates here with a visual language that can be interpreted, based on fundamentally allegoric images.

Due to the surge in these phenomena and the overflow in the use and creation of images, new studies and didactic proposals arise which try to give an answer to a subjectivity caused by a society that is completely mediatized, as can be seen in several papers such as those of Marcos-Dipaola (2019), Corrales (2018), Aguado and Villalba (2020), Ortega Caballero (2014), Estrada-Gonzalez et al. (2020), or Ramón-Verdú et al. (2022), where the creation of photographic images, in situations of sanitary confinement is studied in order to transmit visual messages about feelings and moods. This trend is in full progression, encouraged by communication of the masses, supported mainly by the use of the Internet and *a la carte* contents to be used, *via* platforms of audiovisual dissemination and those of videogames (Fanjul-Peyró et al., 2019), all of which exceed the individual's capacity of assimilation (Andrade-Vargas et al., 2021).

At this moment in time, in the themed context of visual education, with multiple studies and theories, on which this investigation centers, with the interest focused on young university students studying a teaching degree in primary education, young people who are in very close contact with the image, as both users and creators. Work by authors, such as Marín-Viadel (2005, 2011), Acaso López (2009); Acaso López et al. (2011), Hernández (2010), and Huerta (2021a,b), have been taken as reference, papers in which studies have been carried out for the development of language and visual culture in educational contexts, with the photographic and artistic image as a connecting thread. It is worth pointing out, at this stage, that in the future profession of the teacher training students who are the object of study in this paper, the image and language will be a first-level resource for them, and they will be able to use it to educate their students and identify a range of situations in them. With respect to this, the use of the image to discover aspects such as its use and creation is also investigated, placing emphasis on the level of reflection that takes place, and the grade of veracity that is given to the message in a system of dialogue and transmission, of both explicit and implicit ideas and messages. In this sense, the main objective is to learn what the relationship that students establish with visual language is—the use of photographic creation, examining this relationship from the students' facet of potential creators and also as a consumer of these images.

A social context that is so mediatized, as is the current one, encourages the dynamic exchange of personal stories being spread *via* technological devices. This means that the user has to be in contact with others in a flexible and dynamic way too, faced with a huge variety of graphic and visual dissemination means and formats, of highly manipulative messages created by the users themselves or by professionals with objectives that may not always be very clear. The creation and reading of visual messages are interpreted here as a need to confront visual language, as it is a transporter of ideas and concepts which affect people.

### Investigation Questions

With the aim of delimitating the different elements of interest in the investigation, the study concentrated on: (i) the educational specialty studied, the gender and age of the surveyed people; (ii) the visual dialogue through the creation and use of images, with the follow-up interpretation and questioning about the veracity of the message. With these nuclear questions as a base, the following investigation questions were drawn up:

- From the premise of the use of visual language by university students, and taking into account the educational specialty being studied, are there significant differences as regards the creation and use of visual messages? The aim is to delve deeper into the possible differences, depending on whether one has an interest in one particular type of study being undertaken or another.
- Given the gender of the students, is there a difference, with respect to the use and creation of related images which are given a visual language, and their social participation? The objective is to see if there is a difference in attitude between genders with respect to the social function of visual language through images.
- Due to the different ages in which studies related to education tend to be carried out, is there a difference between the age of the students and their interests when it comes to visual communication, whether this is for the creation, use, or interpretation of the messages? It is of interest to know if there are particular variations per age range when it comes to the creation or interpretation of the visual message.

## Materials and Methods

The study was carried out in two phases, the first with a transversal study based on a typical individual objective performance target questionnaire. In the second phase, a factorial Principal Components Analysis (PCA) was used to investigate the possible underlying variables there were in the questionnaire, which would give an answer to the fundamental questions of the investigation, which would then be followed by non-parametric tests, so that the hypothesis test would be validated, or not, which would show significant differences from which conclusions could be drawn.

### Description of the Sample

The study group was made up using convenience sampling of a group of students from the subject of “Development of visual and plastic language” in the degree course of Primary school teacher training at the University of Murcia. The selection criteria were defined by non-probability incidental sampling (Vieytes, 2004), as the sample was chosen intentionally, given that there was easy access to the sample for a particular, temporary period of time (Sabariego and Bisquerra, 2009). From an annual number of 393 people, the sample (*N* = 321) represents 81,67% of the people in the fourth year of the previously mentioned degree course, for the academic year 2020–2021, a number considered to be a representative percentage. The sample was distributed among eight different degree specialties of the degree course: Audición y Lenguaje (AL), Educación Física (EF), Educación Intercultural (EI), Educación Musical (EM), Lengua Extranjera Francés (LEF), Lengua Extranjera Inglés (LEI), Pedagogía Terapéutica (PT), and Recursos Educativos para la escuela y el Tiempo Libre (RE).

As regards gender, there were 257 women in the sample group (80.1%), 62 were men (19.3%), and 2 from the gender other (0.6%). In terms of age, the sample consisted of 297 between the ages of 20 and 25 (92.5%), 10 between 25 and 30 (3.1%), 5 between 30 and 35 (1.6%), and 9 older than 35 (2.8%). The distribution of the sample, depending on the specialty being studied was as follows: AL (*N* = 28; % = 8.7), EF (*N* = 49; % = 15.3), IE (*N* = 38; % = 11.8), EM (*N* = 24; % = 7.5), LEF (*N* = 28; % = 8.7), LEI (*N* = 53; % = 16.5), PT (*N* = 52; % = 16.2), and RE (*N* = 49; % = 15.3).

### Instrument

Data was gathered by means of a questionnaire with an ordinal number scale or four levels, attributing values of 1 (not at all), 2 (some), 3 (quite a lot), and 4 (a lot). It was structured using four blocks of questions: In the first block (*N* = 3), sociological-type questions were asked with context variables (specialty, gender, and age). The second block investigated the use of the photographic image (*N* = 8), the third the graphic image (*N* = 6), and the fourth was used to obtain answers related to the interpretation of the images used (*N* = 7). The validation of the instrument was done by putting it before five experts in the area being investigated, using a template of qualitative evaluation with the areas of sufficiency, clarity, coherence, and relevance of the proposed questions (Urrutia et al., 2014). Several questions were modified, and the pilot test was carried out (*N* = 35; NC = 90%; Q = 5%), obtaining a uniform, coherent set of questions being dealt with (See instrument in Supplementary Material).

### Procedure

The instrument was used on students in their fourth year of the degree, at a time considered ideal, given the intellectual maturity that could be attributed to them. The instrument was given to them *via* a link, before the teaching of the subject actually began, avoiding thus previous conditioning of the questions. Tacit consent was obtained from the participants once they had been informed of the procedure as well as the aim of the investigation, which was filled in freely within the allocated time. Once this had concluded, the data was downloaded to be later analyzed.

### Data Analysis

Analysis was conducted with the program used for statistical analysis IBM SPSS Statistics 28 for Windows. The Kolmogorov-Smirnov test acted as a normality test, showing that the data do not follow a normal distribution, achieving a result of *p* < 0.05 in all variables. The reliability of the construct was checked with an analysis of the internal consistency with the Alfa de Cronbach test, giving coefficient reliability of α = 0.807; *n* = 20, once questions of sociological background and a question of a qualitative nature have been excluded (P8).

The validity of the construct was performed using the factorial analysis of principal components (PCA), to check the feasibility of factorial analysis. By using the sample suitability test Kaiser-Meyer-Olkin (KMO = 0.801) and the Bartlett (*p* < 0.005) sphericity test, it was shown that existing correlations between items did not constitute a matrix identity, showing statistically significant data and therefore a null hypothesis could be ruled out (H0).

For component rotation, the oblique rotation method Direct Oblimin was used with a Kaiser normalizer, as it was considered that there was some correlation between variables. This action tends to reveal a structure that facilitates data interpretation (Lloret-Segura et al., 2014), besides showing evidence of the underlying latent variables, which can give way to a new distribution of the reactive of the instrument, so as to explain the relationships there are between the categorical context variables and the components extracted. The factorial scores were kept as regression variables.

## Results

The PCA revealed six components with their own values of > 1, which explained the 60.053% of the accumulated variance, with a percentage of variance in each of the components as follows: C1 = 23.400; C2 = 11.217; C3 = 7.404; C4 = 6.68; C5 = 5.867; C6 = 5.485. After 10 iterations, the rotated Oblimin matrix component gave a new distribution, becoming conceptually configured depending on the function of the component and the reactive, in the order of the value of its variance in the following way: C1 = P1, P9, P11, P10, P2; C2 = P21, P16, P19, P20; C3 = P6, P5, P3, P7, P4; C4 = P18, P17; C5 = P14, P13, P12; C6 = P15.

The new distribution provided by the PCA shows a new alternative grouping, allowing for a new interpretative focus to be given to the instrument. This organization suggests a change in the initial naming, where C1 becomes: Use of professional and recreational visual messages, for grouping issues related to the use of images for their profession and personal recreational interests; C2: Grade of interpretation of the visual message, for grouping issues related to the interpretation of the visual message; C3: Grade of transmission of the image and one's own feelings, as they are related to the emission of visual messages of a personal nature; C4: Value of privacy of one's own and others' visual image, by grouping those related to giving or requesting permission to use images; C5: Grade of creation of professional and recreational visual messages, for grouping those related to the creation of visual messages related to their profession, RRSS and personal concerns; C6: Veracity of the visual message, related to the reality represented in the visual message.

In Table 1 the descriptive statistics of the instrument can be observed after the recoding of variables. Component 4 stands out due to the marked dispersion of data (*SD* = 1.01) and particularly marked mesokurtic kurtosis (*g*_2_ = −0.88), which could indicate, as a whole, diversity in the answers. Component 2 also stands out somewhat because it is the one with the least dispersion (*SD* = 0.583; σ = −0.155; *g*_2_ = 0.218) with relatively low asymmetry and kurtosis.

**Table 1:** Descriptive statistics of recoded variables.

**Comp**.	**Name**	**No. items**		***Sd***	**σ**	***g_**2**_***
1	Use of professional and recreational visual messages	5	2.850	0.624	0.117	−0.506
2	Grade of interpretation of the visual message	4	2.841	0.583	−0.155	0.218
3	Grade of transmission of the image and one's own feelings	5	3.112	0.684	−0.205	−0.650
4	Value of privacy of one's own and others' visual image	2	2.866	1.011	−0.477	−0.880
5	Grade of creation of professional and recreational visual messages	3	2.489	0.707	0.199	−0.222
6	Veracity of the visual message	1	2.386	0.612	0.360	−0.029

### Comparative Components-Variables of Context

To check if there were significant differences in each of the areas studied, comparatives were carried out between the components and the context variables, in order to accept or reject the H0 null hypothesis: the distribution of component data was the same in all categories; H1: the distribution of component data was not the same in all categories. This comparative was done with the non-parametric Kruskal-Wallis *H*-test for several independent samples (α < 0.05; level of reliability = 95%), taking the six extracted components as test variables, and the context variables of Specialty, Gender, and Age as group variables.

**Figure 1:**
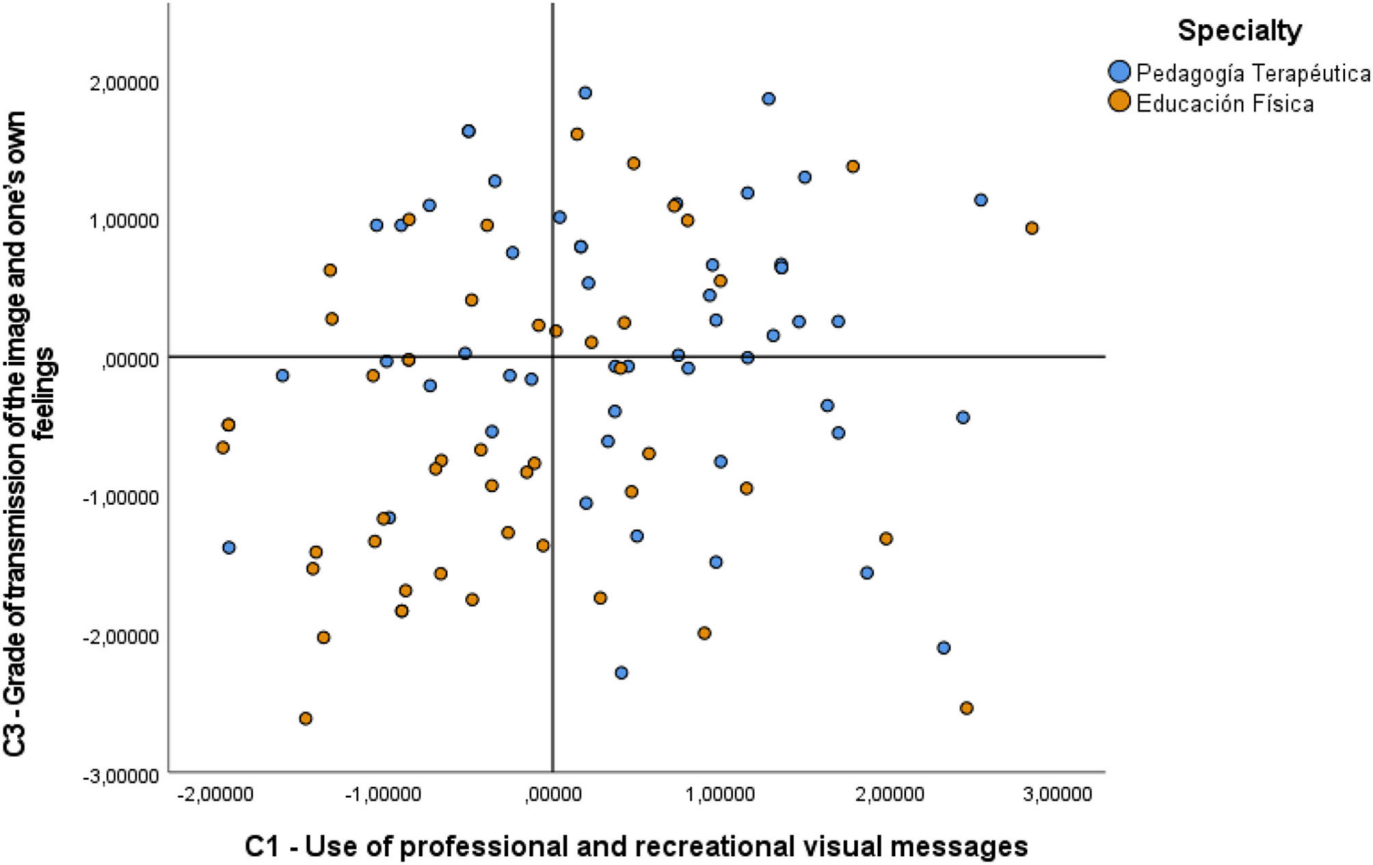
Comparative C1 and C3 with specialties RT-EF.

As can be observed in Table 2, the *H* statistics of the components and the specialty group variable showed significant differences in Component 1 (*p*-value = 0.004; *X*^2^ = 20.921), in Component 3 (*p*-value = 0.029; *X*^2^ = 15,579), in Component 4 (*p*-value = 0.006; *X*^2^ = 19.946) and in Component 5 (*p*-value = 0.021; *X*^2^ = 16.486), therefore, in these mentioned components the null hypothesis (H0) for each of the group variables indicated was rejected. The *post hoc* comparatives in Kruskal-Wallis in Component 1 showed significant differences between the specialties PT-EF (*t* = −68.181; sig. adjust. = 0.006), PT-LEI (*t* = −63.963; sig. adjust. = 0.012), and PT-EI (*t* = −62.407; sig. adjust. = 0.046). In Component 3, significant differences were found in the comparatives of the specialties EF-PT (*t* = −58.39; sig. adjust. = 0.044) and between EF and RE (*t* = −65.204; sig. adjust. = 0.014), differences which can be observed in the graph in Figure 1. This figure shows only the PT-EF comparisons because they coincide in components C1 and C3. Here, in C1, EF shows a lower dispersion of data and more negative values with respect to PT, as in C3, where PT also shows a greater positive trend and a lower dispersion that does not exceed value 2 in the graph.

**Table 2:** Kruskal-Wallis *H*-test statistics: variables of test-group.

	**Component**					
	**1**	**2**	**3**	**4**	**5**	**6**
**Group variable: speciality**						
Chi-squared	20.921	4.047	15.579	19.946	16.486	10.205
gl	7	7	7	7	7	7
Asymptotic sig.	0.004	0.774	0.029	0.006	0.021	0.177
**Group variable: gender**						
Chi- squared	8.327	0.183	28.875	6.682	0.178	4.529
gl	2	2	2	2	2	2
Asymptotic sig.	0.016	0.912	<0.001	0.035	0.915	0.104
**Group variable: age**						
Chi- squared	13.801	4.908	23.022	1.864	1.159	3.876
gl	3	3	3	3	3	3
Asymptotic sig.	0.003	0.179	<0.001	0.601	0.763	0.275

**Figure 2:**
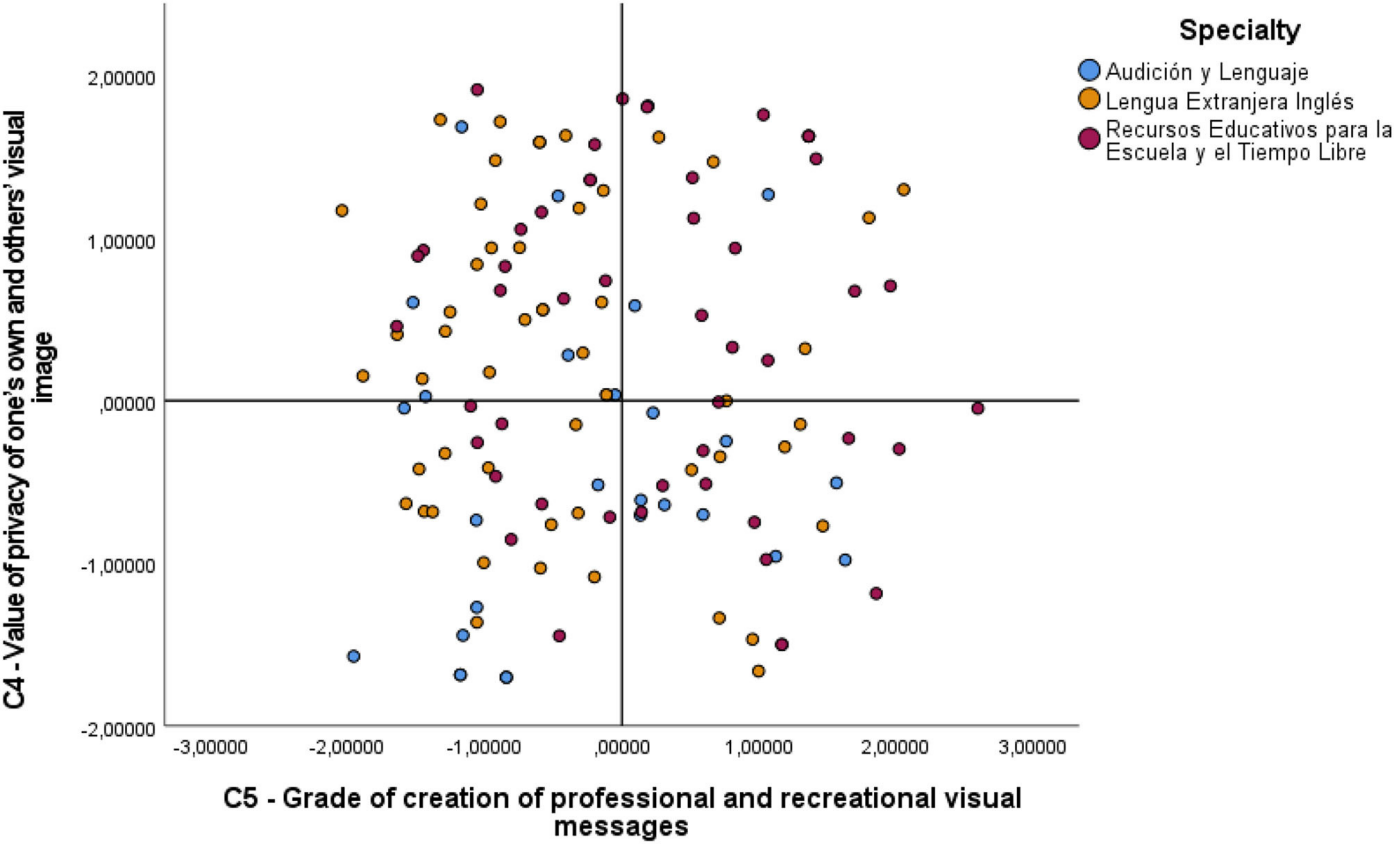
Comparative components 4 and 5 with specialties AL, LEI, and RE.

As regards Component 4, a significant difference was seen in the comparison between the AL-RE (*t* = −68.235; sig. adjust. = 0.047), and finally, in Component 5 there was a significant difference between the specialties LEI-RE (*t* = −60.016; sig. adjust. = 0.031), all of which had significance values of <0.05. These differences can be observed in the graph in Figure 2, where C4 and C5 are shown in function of the specialties that these significant differences represent. Here we can see in C4 a clearly negative trend in AL with respect to RE, where positive values predominate, and in C5, a clearly greater grouping of LEI in negative values, with respect to RE, with much more dispersed values.

**Figure 3:**
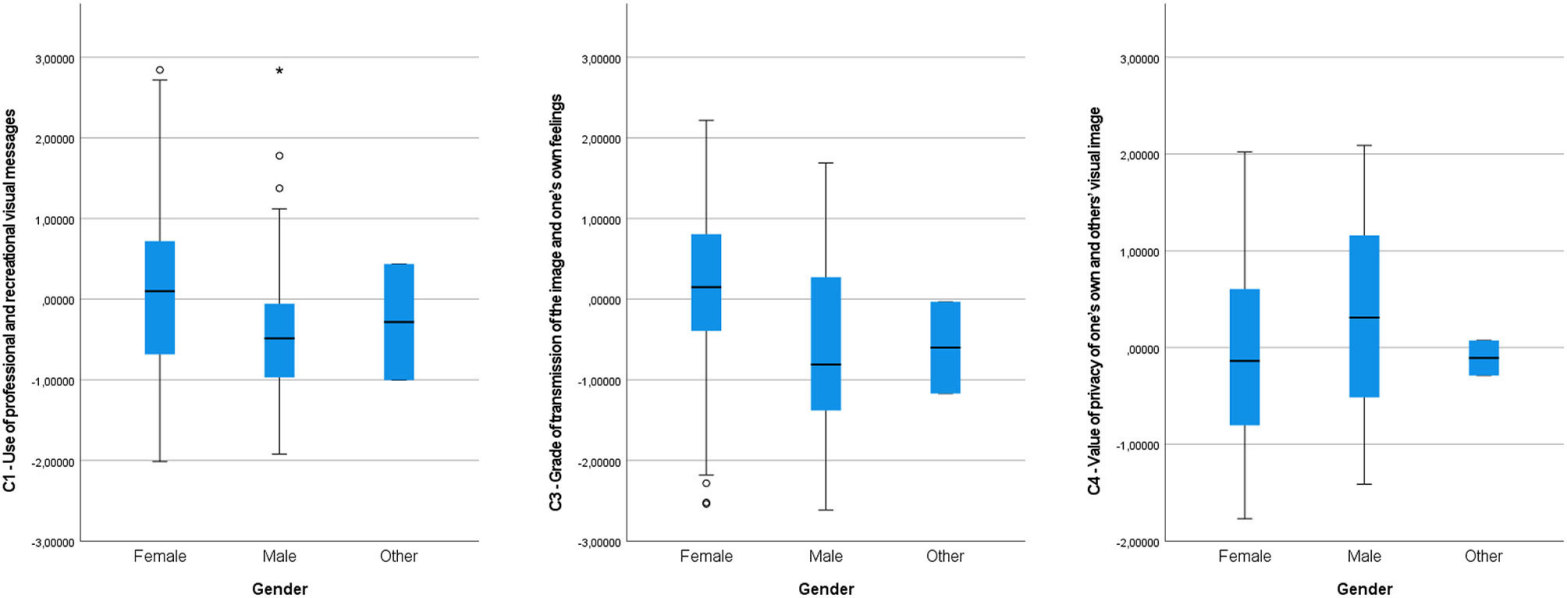
C1, C3, and C4 comparative diagrams in the variable category gender.

The statistics of the H-test of Table 2 between the Components and the group variable Gender, showed significant differences in Component 1 (p-value = 0.016; X^2^ = 8.327), and in Component 3 (p-value < 0.001; X^2^ = 28.875) and in Component 4 (p-value = 0.035; X^2^ = 6.682), thus, in the mentioned components, the null hypothesis (H0) was rejected. In the *post hoc* analysis, in Component 1 significant differences could be seen between men and women (t = 37.459; sig. adjust. = 0.013), and in Component 3 of the same duality (t = 69.403; sig. adjust. = 0.000), and the same thing occurred in al Component 4 (t = −33.942; sig. adjust. = 0.029), with the significance values level of all of these at <0.05, as can be observed in the diagrams in Figure 3, however, in the other gender, no variations are observed, because it is a very small sample, with data that may not be representative.

**Figure 4:**
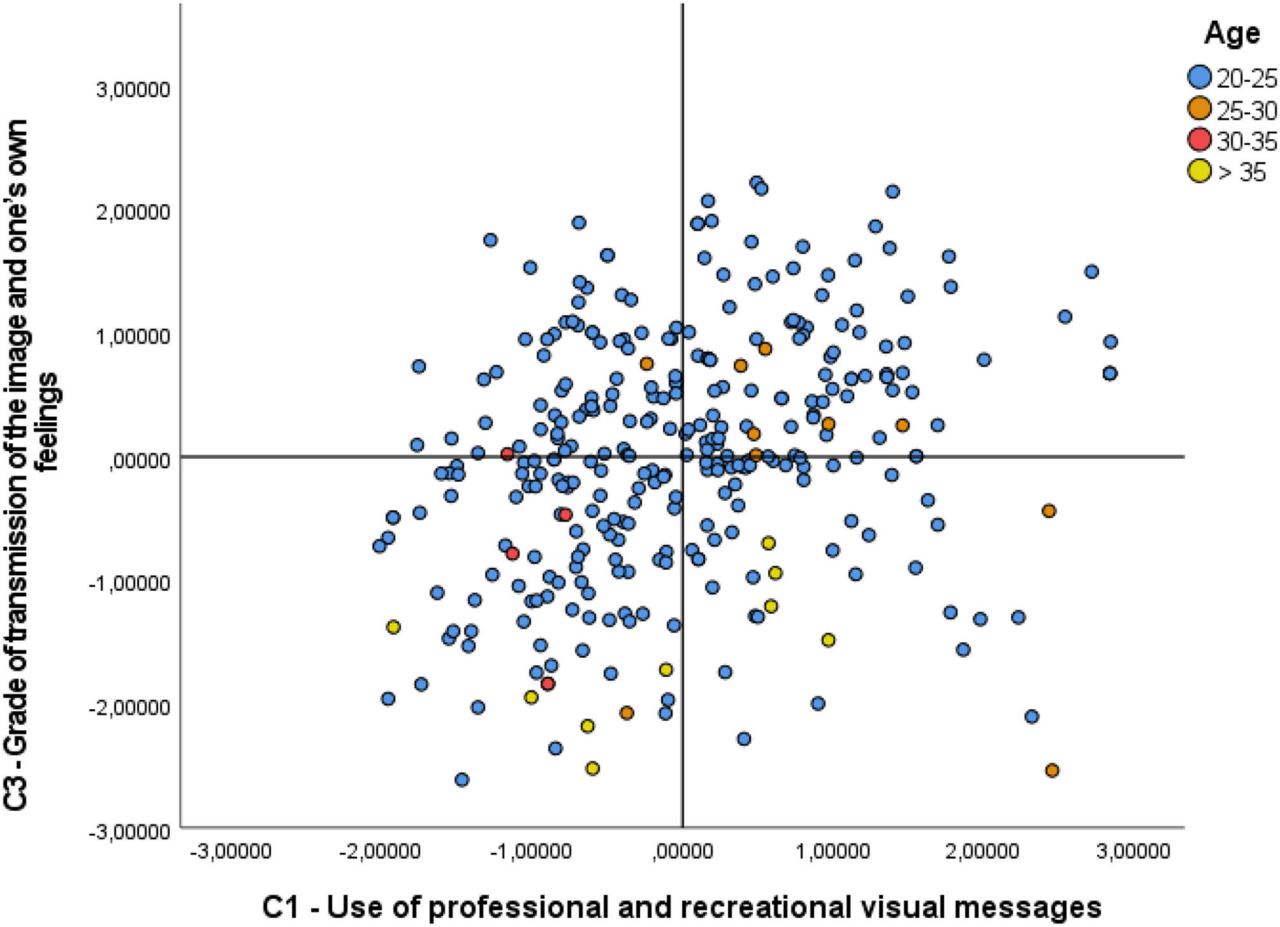
Comparative components 1 and 3 with age.

As for the statistics of the Kruskal-Wallis H-test, between the Components and the group variable Age (Table 2), significant differences showed up in both Component 1 (p-value = 0.003; X^2^ = 13.801) and Component 3 (p-value < 0.001; X^2^ = 23.022), and as a result, the (H0) null hypothesis is rejected in the mentioned components. In the *post hoc* analysis, Component 1 showed differences only between the age ranges 30–35 and 25–30 (t = 186.1; sig. adjust. = 0.002). In Component 3, the differences were found between two age ranges: Over 35 and 25–30 (t = 126.022; sig. adjust. = 0.019), and over 35 and 20–25 (t = 134.731; sig. adjust. = 0.000), taking, once again, as a level of signification, values of <0.05. In Figure 4 the distribution of the C1 and C3 values can be seen on the graph, in the function of the age ranges and the aforementioned significant differences. Visually, in C1 the negative values in the 30–35 age range clearly stand out, as opposed to the positive ones in the 25–30 age range. In C3, large differences are observed between those over 35, with clearly negative values, with respect to the 25–30 age range, mostly positive, as well as with the 20–25 age range, which, although they cover all the ranges of the graph, positive values with little dispersion predominate.

## Discussion and Conclusion

As demonstrated by this and other studies mentioned, the consumption and creation of images in our society is a topic of concern to numerous researchers, particularly those related to education, as is the case of the study by Ramón-Verdú et al. (2022), where fundamental aspects about their behaviors and communication through images in teachers in training are questioned, or also, the work of Aguado and Villalba (2020), where the consumption of illustrations in the classroomsof Early Childhood and Primary Education is studied. However, in this sense, we did not find current studies that directly analyze the consumption and production of images in university students as future teachers in training. We did find other closely related research, such as the study by Andrade-Vargas et al. (2021), which analyzes the behavior and use of social networks in young people, where the image plays a priority role in the current communicative model, as well as the studies by Mesquita-Romero et al. (2022) and Marzal-Felici and Soler-Campillo (2011) where research is conducted on literacy and critical sense about the image coming from digital and media environments, as well as the consumption habits and uses of photography by students in the digital era. These are some of the references in which the research carried out is contextualized, analyzing behaviors and showing relevant and closely related data.

The data results of the analysis reveal certain behavioral differences depending on the premises raised at the beginning of this document. The tests carried out were done so in a special context due to, on the one hand, the social isolation that took place because of the world pandemic, and on the other, the university context in which the investigation was focused. This context has special characteristics as the students form part of a social network made up of educational professionals. This means that the use and limitations of visual language in contexts of development, where that which is visual is predominant, depends on the command one has of the area in question, and the importance lent to visual language. The capabilities as regard the mastery of visual language will very much constitute the basis for the construction of the professional personality of the future teacher, putting creativity and an artistic vision of education into practice. This is the domain in which the ability to dialogue through the use of images in digital spaces of social confluence is built and developed, places that abound with accurate visual information, but where there are also misleading messages, with very different aims.

With respect to the first premise made, the possible differences depending on the creation and use of images in professional profiles of the specialties, there are differences in several of the components extracted. As regards Component 1, and its relationship with the use of professional and recreational visual messages, the specialty which stands out most is that of PT, as there are clear differences between this and the other specialties of mainly EI, LEI, and EF. As far as we are concerned, this is a reflection of greater interest by PT students in using images that act as support when intervening with children with special needs, given the overriding value that this specialty has to provide corrective solutions. It seems that the use of images on a professional level or for recreational purposes is of great help in teaching which is of a therapeutic nature. This, however, does not happen in the EF specialty, where there is least usage of visual messages on a professional or recreational level, due to, in our opinion, the fact that, in the field of sport, although images are used, these are of a documentary-type, more so than those that can be used as transmitters of messages. When it comes to the specialties that are not mentioned (LEF, EI, AL, and EM), there are no significant differences.

**Figure 5:**
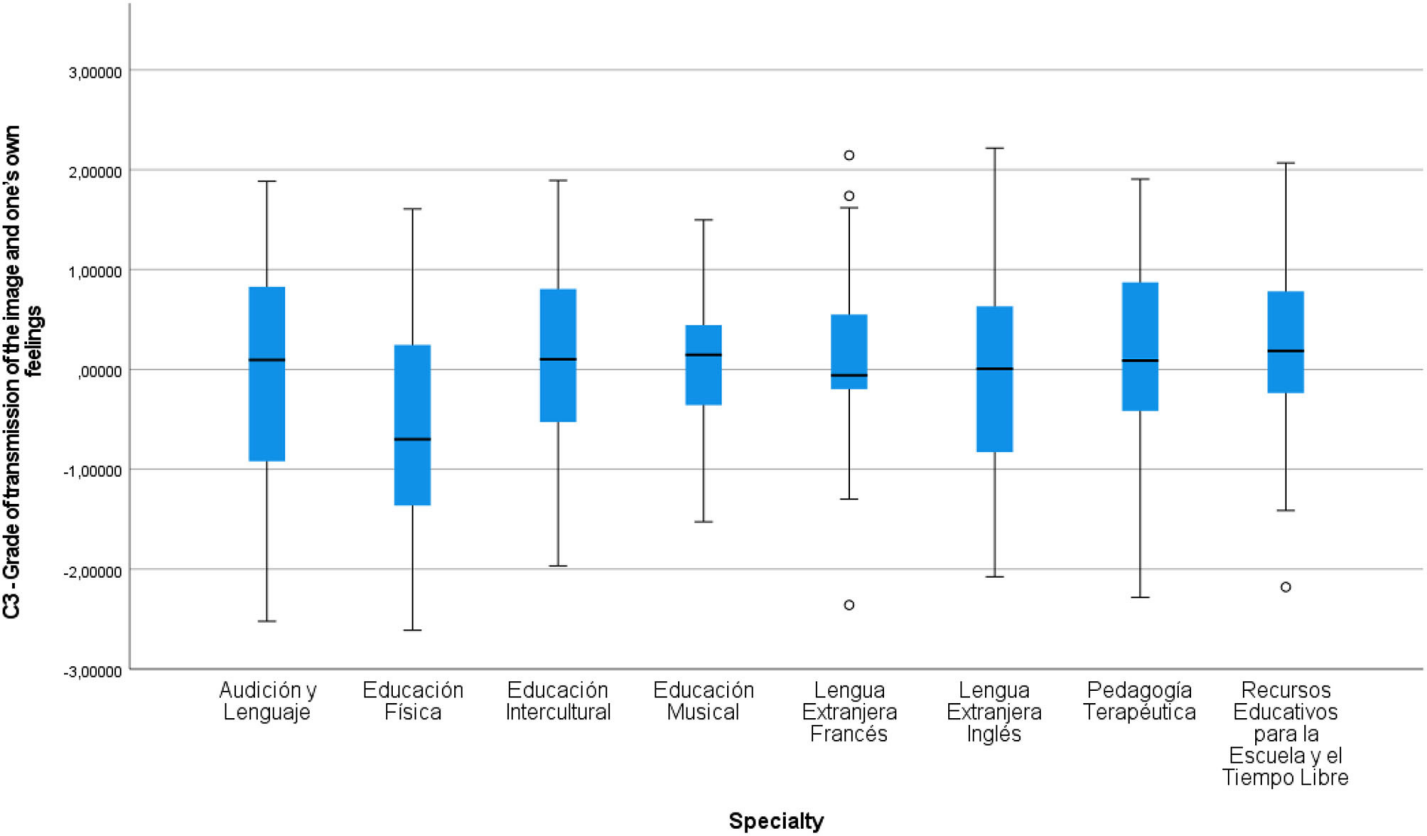
Kruskall-Wallis *H* for independent samples. Comparative C3-specialities.

With respect to this first premise and Component 3, which is related to the grade of transmission of one's own feelings and the image, the differences shown stand out in EF as opposed to PT and RE, with those of EF being clearly less. It seems that students of this specialty are less interested in transmitting their own images or images which reflect feelings. Despite being a specialty that transmits values, this could be interpreted as that the students are less interested in transmitting feelings, less so than in other specialties, as the socialized physical improvement dominates over other educational aspects, which are, nonetheless, also present. In the rest of the specialties, no particular differences worth pointing out were detected. Graphically-speaking, the different values arising from the Kruskall-Walllis *H*-test in the C3 comparative and the specialties, can be seen in Figure 5.

As for Component 4, which concerns the value given to the privacy of one's own and others' image, and the first premise, the only difference detected was that in the AL and RE duality. In the first specialty, it could be seen that privacy was valued a lot less than in the second specialty. We believe that this is due to the fact that in the second specialty, education is of a more recreational nature, where the most are made of the educational time available, which, obviously leads to graphic creations in situations that are highly expressive and shared. Finally, in Component 5 and the level of creation of professional and recreational visual messages, the comparative which stands out most is that of LEI-RE, where, just like Component 4, the second specialty (RE), is where there is most participation, a lot more than the first. Once again, in the RE specialty, in which education takes place in a more recreational way, the creation of images takes a more important role, contrary to that in LEI.

With regards to the second premise, which refers to whether there are differences within genders in the attitude demonstrated toward the social function of visual language through images, differences were seen between Components 1, 3, and 4. The first, which refers to the use of professional and recreational images, is clearly superior in women to men. The level of transmission of one's own image and feelings is still less among men in comparison to women. It is our opinion that this may be due to the fact that the preconceptions of the masculine stereotype may still exist and the refusal to show feelings or to avoid comparisons about their own image, also. This fact may be in relation to the C4-Gender comparative, as in this case, it is the male gender that is more reserved when it comes to showing its own image or using that of others, placing greater value on privacy.

On analysis of the third premise, that of the variability of behaviors regarding the visual message depending on age, significant differences were shown in two of the components. In Component 1, this difference could be seen in the age ranges of 30–35 and 25–30. The first range revealed a lower level of use of visual messages of a professional or recreational level, as opposed to much higher use in the age range of 25–30 (Figure 5). It is our belief that this may be due to the maturity level of the participant and there being less interest in using recreational-type images to share on social networks, which is something that does not occur in the age range 25–30, this being the natural age of full social vibrancy. In Component 3 there are differences between the age ranges above 35 and 25–30, as well as above 35 and 20–25. In this case, the level of transmission of one's own image or feelings is extremely low. At this age, there is a natural loss of interest in achieving social recognition, and also, active participation in social networks has decreased enormously, where the need for recognition of the self in social groups that one forms part of, fades, as personal and intellectual maturity has normally been reached. Images that reflect moods, or “selfie” images in which one directly shows one's physical aspect are quite marked in the age range 25–30, but even more so in the 20–25 age range, which is an age in which one actively looks for support and recognition, socially.

It is important to highlight the fact that Component 2: Level of interpretation of the visual message did not show significant disparities in any of the analyses carried out. This shows that the interest in the interpretation of the message transported by the images is very alike in all the context variables, showing a certain interest in the search for the meaning of the message. On the other hand, as regards Component 6: Veracity of the visual message, where the level of veracity attributed to the message is measured, even though there were no significant differences, the valuations are clearly of a negative kind. These results reaffirm the studies done by Beneyto (2021) or Marzal-Felici and Soler-Campillo (2011), in relation to the semantics of the image and the reality it shows, and the attribution of the value and lack of credibility of journalistic photography.

The results show, generally speaking, considerable differences when it comes to the use of the image as an instrument for transferring meanings. There are differences as regards ages, in the aspect of both the user and the creator of the images where the older age groups, although they value these aspects more, they use them less. There is also evidence among the genders, as men tend to be more reluctant to lend their image to others and to show feelings which, as far as we are concerned, could show vulnerability, something that does not happen among women.

What this investigation has come up with constitutes the ratification and the starting point from which future educational topics of interest within the ecosystem of visual culture, something which is more and more present, and to a greater degree in a mediatized society, can be confronted. The differences detected between groups show the need to carry out future training action in youth, who can provide the mastery and control of what is visual, and where, more than ever, certain styles of communication that camouflage the truth by manipulation, are becoming the norm. We believe that visual language makes up one of the universal languages which need to be investigated further. The investigation of the meanings, messages, and formats is a real challenge nowadays, given the dynamicity and versatility that the Internet and social networks promote. In general terms, there must be a command of this language so that, from the point of view of future teachers, it can be established as a habitual, quality system of communication.

## Data Availability Statement

The original contributions presented in the study are included in the article/Supplementary Material, further inquiries can be directed to the corresponding author.

## Author Contributions

All authors listed have made a substantial, direct, and intellectual contribution to the work and approved it for publication.

## Conflict of Interest

The authors declare that the research was conducted in the absence of any commercial or financial relationships that could be construed as a potential conflict of interest.

## Publisher's Note

All claims expressed in this article are solely those of the authors and do not necessarily represent those of their affiliated organizations, or those of the publisher, the editors and the reviewers. Any product that may be evaluated in this article, or claim that may be made by its manufacturer, is not guaranteed or endorsed by the publisher.
