# Supplementary material for: The Image as Language: The Creation and the Use of the Visual Message by Young University Students in Their Communicative Social Activity
Source: Front Psychol. 2022 Jul 7;13:944187. doi: 10.3389/fpsyg.2022.944187 (PMC9302490; doi:10.3389/fpsyg.2022.944187)
Supplement: Supplementary file 1 [file Data_Sheet_1.pdf]

INSTRUMENTO

**CUESTIONES DE TIPO SOCIOLÓGICO**	
Nombre y apellidos del estudiante	
Mención que cursa	Audición y Lenguaje<br>Educación Física<br>Educación Intercultural<br>Educación Musical<br>Lengua Extranjera Ingles<br>Lengua Extranjera Francés<br>Pedagogía Terapéutica<br>Recursos Educativos para la Escuela y el Tiempo Libre
Género	Masculino<br>Femenino<br>Otro
Edad (en años)	20-25<br>25-30<br>30-35<br>Mayor de 35

	**DIMENSIÓN 1: USO DE LA IMAGEN FOTOGRÁFICA**	
		Nada – Algo – Bastante - Mucha
1	¿En qué grado te consideras consumidor de fotografías relacionadas con la educación?	
2	¿En qué grado utilizas fotografías relacionadas con tus aficiones?	
3	¿Con qué frecuencia realizas fotografías para documentar momentos de ámbito social y lúdico?	
4	¿Con qué frecuencia realizas fotografías artísticas?	
5	¿Con qué frecuencia realizas selfis?	
6	En relación con la anterior, ¿qué importancia le das a la imagen que proyectas hacia los demás mediante la fotografía?	
7	¿Utilizas la fotografía para expresar algún tipo de sentimiento o circunstancia como, por ejemplo: alegría, tristeza, belleza, fuerza, logro, superación o reto?	
8	*En relación a la pregunta anterior, ¿cuál de los siguientes destacarías?*<br>*Alegría, Tristeza, Belleza, Fuerza, Logro, Superación, Reto (Escríbelo si no está en la lista)*<br>**Nota**: esta pregunta, por ser de naturaleza cualitativa, no se ha utilizado en el análisis estadístico.	

	**DIMENSIÓN 2: USO DE LA IMAGEN GRÁFICA**	
		Nada – Algo – Bastante - Mucha
9	¿En qué grado utilizas imágenes gráficas (dibujos o ilustraciones) relacionadas con la educación?	
10	¿En qué grado utilizas imágenes gráficas (dibujos o ilustraciones) relacionadas con tus aficiones?	
11	¿En qué grado utilizas imágenes gráficas (dibujos o ilustraciones) relacionadas con tu contexto profesional?	
12	¿Con qué frecuencia creas imágenes gráficas (dibujos o ilustraciones) para trabajos académicos?	
13	¿Con qué frecuencia creas imágenes gráficas (dibujos o ilustraciones) para mostrar a los demás, fruto de tus inquietudes o intereses?	
14	¿Con qué frecuencia creas imágenes gráficas (dibujos o ilustraciones) para mostrar en redes sociales o aplicaciones de mensajería instantánea (tipo WhatsApp)?	
	**DIMENSIÓN 3: REFLEXIÓN Y VERACIDAD**	
		Nada – Algo – Bastante - Mucha
15	¿Crees que las fotografías que habitualmente ves o utilizas reflejan la realidad de lo que representan?	
16	¿Con qué frecuencia sueles reflexionar acerca del mensaje contenido en las imágenes que ves en tus redes sociales o en Internet?	
17	¿Pides permiso a la gente que etiquetas en las imágenes que subes a las Redes Sociales? No contestes en caso de no tener red social.	
18	¿Sueles dar permiso para que te etiqueten en una imagen en una red social? No contestes en caso de no tener red social.	
19	En cuanto a la publicidad, ¿con qué frecuencia sueles reflexionar acerca del mensaje contenido en las imágenes publicitarias, ya sea fotografía o video?	
20	¿En qué grado sueles buscar la veracidad en las imágenes que ves, por ejemplo, contrastando la fuente de la que procede la imagen con otras fuentes?	
21	¿En qué grado sueles detectar intentos de manipulación en las imágenes que ves, ya sea fotografía o video?	
